# Inhibition of PRL-2·CNNM3 Protein Complex Formation Decreases Breast Cancer Proliferation and Tumor Growth*

**DOI:** 10.1074/jbc.M115.705863

**Published:** 2016-03-11

**Authors:** Elie Kostantin, Serge Hardy, William C. Valinsky, Andreas Kompatscher, Jeroen H. F. de Baaij, Yevgen Zolotarov, Melissa Landry, Noriko Uetani, Luis Alfonso Martínez-Cruz, Joost G. J. Hoenderop, Alvin Shrier, Michel L. Tremblay

**Affiliations:** From the ‡ Rosalind and Morris Goodman Cancer Research Centre, Montréal, Québec H3A 1A3, Canada,; the Departments of § Biochemistry and; ¶ Physiology, McGill University, Montréal, Québec H3A 0G4, Canada,; the ‖ Department of Physiology, Radboud Institute for Molecular Life Sciences, Radboud University Medical Center, 6500 HB Nijmegen, The Netherlands, and; the ** Structural Biology Unit, Center for Cooperative Research in Biosciences (CIC bioGUNE), Technology Park of Bizkaia, 48160 Derio, Bizkaia, Spain

**Keywords:** cancer, cell proliferation, magnesium, patch clamp, phosphatase, Bateman module, CNNM3, PRL-2, thienopyridone

## Abstract

The oncogenic phosphatase of regenerating liver 2 (PRL-2) has been shown to regulate intracellular magnesium levels by forming a complex through an extended amino acid loop present in the Bateman module of the CNNM3 magnesium transporter. Here we identified highly conserved residues located on this amino acid loop critical for the binding with PRL-2. A single point mutation (D426A) of one of those critical amino acids was found to completely disrupt PRL-2·human Cyclin M 3 (CNNM3) complex formation. Whole-cell voltage clamping revealed that expression of CNNM3 influenced the surface current, whereas overexpression of the binding mutant had no effect, indicating that the binding of PRL-2 to CNNM3 is important for the activity of the complex. Interestingly, overexpression of the CNNM3 D426A-binding mutant in cancer cells decreased their ability to proliferate under magnesium-deprived situations and under anchorage-independent growth conditions, demonstrating a PRL-2·CNNM3 complex-dependent oncogenic advantage in a more stringent environment. We further confirmed the importance of this complex *in vivo* using an orthotopic xenograft breast cancer model. Finally, because molecular modeling showed that the Asp-426 side chain in CNNM3 buries into the catalytic cavity of PRL-2, we showed that a PRL inhibitor could abrogate complex formation, resulting in a decrease in proliferation of human breast cancer cells. In summary, we provide evidence that this fundamental regulatory aspect of PRL-2 in cancer cells could potentially lead to broadly applicable and innovative therapeutic avenues.

## Introduction

Protein tyrosine phosphatases (PTPs)^2^ constitute a large family of enzymes that can exert both positive and negative effects on signaling pathways (1). The phosphatases of regenerating liver 1, 2, and 3 (PRL1–3, also known as PTP4A1–3) are members of the PTP family that are highly expressed in the majority of human solid tumors as well as in hematological cancers (2–4). They are weakly active in *in vitro* enzymatic assays using synthetic substrates, and some physiological substrates have been proposed but remain to be confirmed (5–8). Despite recent publications in the cancer field, the normal physiological functions of PRLs are poorly understood. In our recent work, we uncovered a new paradigm whereby PRLs regulate magnesium transport by forming a complex with the CNNM magnesium transporters to promote breast cancer development (5). Such an association was later reported by Funato *et al.* (9), thus establishing a novel role for PRLs in the regulation of intracellular magnesium levels.

Like protein phosphorylation, the intracellular magnesium balance is altered in transformed cells and has been linked to modifications of several hallmarks of cancer (10). Importantly, high intracellular levels of this metal cation seem to confer a metabolic advantage to cells and to promote acquisition of a transformed phenotype (11–13). In recent years, the existence of novel mammalian genes encoding proteins directly involved in the transport of magnesium through cell membranes has been uncovered (14). Among those is the human Cyclin M (CNNM) gene family comprising four homologs (CNNM1–4) that are differentially expressed in human tissues and are present throughout evolution (15, 16). Genome-wide studies showed that CNNM2-, CNNM3-, and CNNM4-specific single nucleotide polymorphisms were associated with serum magnesium concentrations, supporting the role of these proteins in human magnesium homeostasis under physiological conditions (17).

Secondary structure prediction for the CNNMs suggests the presence of three transmembrane domains located at the N-terminal region and a C terminus containing a cystathionine β synthase (CBS) pair domain (Bateman module) essential for the binding to PRLs via a unique extended amino acid loop absent in other CBS-containing proteins (5, 16). Interestingly, a mutation in the Bateman module of CNNM2 was reported to cause a dominant form of blood hypomagnesemia (18), confirming the importance of this CNNM region in the regulation of magnesium homeostasis. Currently, seven families with CNNM2 mutations suffering from hypomagnesemia have been described (18, 19).

Here we identified a highly conserved residue located in the extended amino acid loop of the second CBS domain of all CNNMs that is critical for the PRL-2·CNNM3 oncogenic function. Furthermore, blocking the formation of this complex using a small molecule inhibitor showed an antiproliferative effect on human breast cancer cells, indicating a potential novel therapeutic avenue to treat cancer patients.

## Experimental Procedures

### 

#### 

##### Conservation of the CNNMs Bateman Module and Modeling of the CNNM3·PRL2 Complex

ConSurf (20) was used to calculate the sequence conservation score of the CNNM2 Bateman module (PDB code 4IY0) and its homologues. The structure figure was rendered using the UCSF Chimera 1.10.2 package (21). Because of the lack of coordinates for CNNM3 and PRL-2 in the databases, the first step for modeling their interaction consisted of constructing suitable three-dimensional templates of the two interacting proteins from their closest homologs (CNNM2, PDB code 4IY4; PRL1, PDB code 1XM2). After substituting the variant residues manually with COOT (22), an optimization of the overall geometry was carried out with PHENIX (23). The protein-protein docking prediction was done with ZDOCK (24). ZDOCK is a fast Fourier transform-based protein-docking program that searches all possible binding modes in the translational and rotational space between two structural models and evaluates each pose using an energy-based scoring function.

##### Plasmid Construction, Transfection, and Pulldown Experiments

Cloning of human PRL-2 and CNNM3 into pDEST15, pDEST17, pLenti6-v5, pcDNA3.1 FLAG, pcDNA3.0 His-FLAG-DEST, and pDEST27 (Open Biosystems, Thermo Scientific) were described previously (5). Point mutations were introduced by site-directed mutagenesis using the QuikChange site-directed mutagenesis kit (Stratagene, Agilent Technologies, Santa Clara, CA). The luciferase reporter gene was also cloned in pMSCV-Puro internal ribosomal entry site GFP, a gift from Scott Lowe (Memorial Sloan Kettering Cancer Center, New York, NY; Addgene plasmid 18751) to produce a pMSCV-Puro internal ribosomal entry site GFP luciferase plasmid. HeLa cells were transfected with various plasmids, followed by either GST pulldown or FLAG immunoprecipitation as described previously (5). HeLa cells were used only as a mammalian expression tool to produce the recombinant protein required for pulldown experimentation.

##### Co-infection in DB-7 and CNNM3 Stable Line Production

DB-7 cells were stably co-infected using the lentiviral vector pLenti6-v5 (Invitrogen) containing the human CNNM3 gene and the retroviral MSCV-Puro internal ribosomal entry site GFP luciferase construct. First, HEK293T/17 cells were transfected with the appropriate lentiviral or retrovirus constructs using Lipofectamine 2000 (Invitrogen). Cultured supernatants were collected 36–48 h after transfection and filtered. DB-7 cells were co-infected with the filtered viral supernatants in a 1:1 ratio in the presence of 4 μg/ml Polybrene for 48 h, after which the medium was changed. Following infection, cells were selected with 5 μg/ml blasticidin and 2 μg/ml puromycin for 2 weeks, and the resistant population was used for cellular assays.

##### Electrophysiology

All currents were recorded using an Axopatch 200B amplifier (Axon Instruments, Sunnyvale, CA) coupled to a CV 203BU headstage in the whole-cell patch clamp configuration. To ensure adequate voltage control, a minimum of 80% series resistance compensation was required along with an access resistance of <10 megohms. Command pulses were generated by a Digidata 1440A controlled by pClamp 10.4 software (Axon Instruments). HEK293 cells are extensively used as an expression tool to study isolated receptor channels and transporters activities (25). The HEK293 cells were clamped at a holding potential of 0 mV, from which 2-s steps were made in increments of 25 mV over the range of −150 to +25 mV, followed by a return to 0 mV. Data were acquired at 20 kHz and low pass-filtered at 2 kHz. Peak current amplitudes were quantified using Clampfit 10.4. All current values were normalized to cellular capacitance (picofarads). Borosilicate glass pipettes (Warner Instruments, Hamden, CT) were prepared with a microprocessor-controlled multistage puller (P97, Sutter Instruments, Novato, CA) to produce a tip resistance of 2–4 megohms when filled with 135 mm KCl, 5 mm EGTA, and 10 mm HEPES (pH 7.2) with KOH (275 mosm). Cells were plated in the perfusion chamber of an inverted microscope (Zeiss Axiovert S100TV) and perfused using a gravity-based flow system (2 ml/min) containing Tyrode solution (135 mm NaCl, 5 mm KCl, 2 mm MgCl, 10 mm HEPES (pH 7.4 with NaOH, 275 mosm)). All experiments were performed at room temperature.

##### Cellular Proliferation

Cell were seeded in a 96-well plate with 5000 cells/well, and quantification was performed at 12, 24, 48, and 72 h using the CyQuant® cell proliferation assay kit (Invitrogen/Life Technologies). For magnesium modulation experiments, cells were allowed to adhere in complete medium for 12 h. After one PBS wash, magnesium-free DMEM (Wisent Inc.) with dialyzed FBS was used to vary the concentrations of magnesium by the addition of MgCl_2_. The relative fluorescence was detected at 485-nm excitation/527-nm emission using a 96-well plate reader (Varioskan Fluostar, Thermo Scientific) and relative fluorescence values were then plotted at each time point. The proliferation of human MCF-7 cells was monitored using a real-time cell analyzer instrument, the xCELLigence system (Roche Applied Science). The cells were seeded in E-plate (Roche Applied Science) with 5000 cells/well. After overnight incubation, various concentrations of inhibitor were added to each well, and the cell index values were monitored at 5-min intervals for the first 6 h and then every 15 min for 96 h. Incubation with dimethyl sulfoxide was also performed as a vehicle control by adding an equivolume of dimethyl sulfoxide compared with the highest inhibitor volume treatment. Three replicates of each concentration of the inhibitor were used for this experiment. The data from the real-time cell analyzer software were exported and analyzed with Prism software (GraphPad, version 6).

##### Cell Surface Biotinylation

HEK293 cells were transfected with wild-type and mutant CNNM3/PRL-2 constructs for 48 h using Lipofectamine (Invitrogen) in 1:2 DNA:Lipofectamine ratio. Subsequently, cell surface proteins were biotinylated as described previously (19). Briefly, cell surface proteins were biotinylated for 30 min at 4 °C in 0.5 mg/ml Sulfo-NHS-LC-LC-Biotin (Pierce). Cells were washed and lysed in lysis buffer (150 mm NaCl, 5 mm EGTA, Triton 1% (v/v), 1 μg/ml pepstatin, 1 mm PMSF, 5 μg/ml leupeptin, 5 μg/ml aproptin, and 50 mm Tris/HCl (pH 7.5)). 10% of the sample was taken as input control, and the rest of the protein lysates were incubated overnight with NeutrAvidin-agarose beads (Pierce) at 4 °C.

##### Colony Formation in Soft Agar

Anchorage-independent growth in soft agar was measured by colony formation assay as described previously (3). The size of each colony was measured using Ilastik image processing tool version 1.1.8 (26). Colonies sizes were then normalized with their respective total colony number and divided into small (50 to <500 pixels), medium (500 to <1000 pixels) and large (≥1000 pixels) categories.

##### Xenograft Assay and in Vivo Bioluminescence Imaging

Mice (NU/J, 6 weeks old) were injected in the no. 4 mammary fat pad with 1 × 10^6^ DB-7 mammary cancer cells stably expressing empty vector, CNNM3-V5, or CNNM3 D426A-V5 as described previously (5). *In vivo* imaging of the injected DB-7 cells also expressing the luciferase reporter gene was performed using a Xenogen IVIS-100 imaging system (Xenogen Inc.). The mice were placed into the imaging chamber of the system 10 min after d-luciferin intraperitoneal injection (150 mg/kg) under anesthesia by isoflurane gas (2%, inhalation). Analysis of the emitted bioluminescence was performed using Live Image Pro. 2.5 software (Caliper Life Science), and fluorescence intensities were expressed as total flux (photons per square centimeter per steradian) were measured by drawing a rectangular region of interest over the entire injection area.

##### In Vitro Pull-down Assay with Thienopyridone

The 16-kDa Bateman module of human CNNM3 (amino acids 302–452) and the 19-kDa PRL-2 construct were expressed in *Escherichia coli* BL21 and purified as described previously (5). Glutathione-Sepharose beads were washed with precooled binding buffer (150 mm NaCl, 20 mm Tris, 1% Triton-X, 1× complete protease inhibitor mixture (Roche), and 5 mm 2-mercaptoethanol), and 20 ng of either purified GST or GST-CBS protein was added to the beads and incubated at 4 °C for 1 h, followed by three washes with the binding buffer. In parallel, an equimolar amount of His-PRL-2 protein (∼10 ng) was distributed in a new set of tubes to which various concentrations of thienopyridone (inhibitor) were added. The His-PRL-2/inhibitor solution was incubated at 4 °C for 20 min and then added to the GST- or GST-CBS-bound beads. This final mixture was incubated at 4 °C for 1 h, and then the beads were washed three times with binding buffer. Bead-bound proteins were boiled in SDS loading buffer before Western blotting analysis.

## Results

### 

#### 

##### Identification of Critical Residues in CNNM3 Is Essential for the Interaction with PRL-2

**FIGURE 1.**
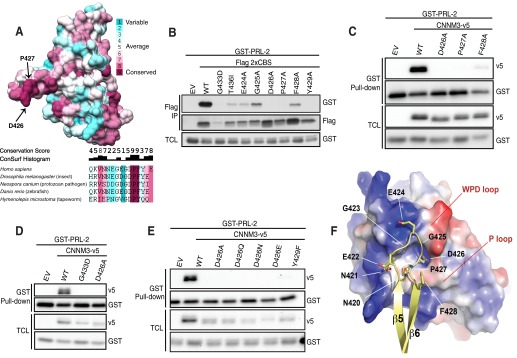
**Aspartate 426 of CNNM3 is critical for complex formation with PRL-2.** *A*, the structure of the Bateman module of CNNM2 is colored based on the residue conservation, ranging from *cyan* (lowest) to *magenta* (highest). The two most highly conserved residues, Asp-426 and Pro-427, are indicated on the CBS loop. The sequence alignments of the amino acid loop from the representative Bateman module of various CNNM homologues are shown in the *bottom panel*. The conservation score and the ConSurf histogram indicate the rate of conservation of a particular residue within 150 homologous sequences. *B*, HeLa cells were transiently co-transfected with GST-PRL-2 and either the tandem CBS domain of CNNM3 or various mutants. FLAG immunoprecipitation (*IP*) was performed on cell extracts, followed by Western blotting analysis with either GST or FLAG antibodies. *EV*, empty vector; *TCL*, total cell lysate. *C–E*, HeLa cells were transiently co-transfected with GST-PRL-2 and either the full-length CNNM3 or various mutants. GST pulldown was performed on cell extracts, followed by Western blotting analysis with either v5 or GST antibodies. *F*, the figure, extracted from the *in silico* model obtained with ZDOCK software, represents the interaction zone between CNNM3 and PRL-2. The surface of PRL-2 is shown in surface representation and colored according to electrostatic potential (positive, *blue*; negative, *red*). The interaction of the extended loop linking the last two β strands of the CBS2 motif of CNNM3 (*yellow ribbons*) with the residues of the catalytic site of PRL-2 or its surroundings occurs mainly through electrostatic forces. The tight turns of the loop are favored by the alternate glycines and proline preceding or following its acidic residues.

We demonstrated previously that a complete deletion of the amino acid loop of the second CBS domain of the Bateman module or a mutation disturbing the full structure of the Bateman module of CNNM3 was able to abolish its interaction with PRL-2 (5). Because the degree to which an amino acid position is conserved during evolution depends strongly on its functional importance, we used the publicly available crystal structure of the CNNM2 Bateman module to determine its conservation among 150 homologous eukaryotic sequences using ConSurf (20) (supplemental Fig. 1). This approach yielded a nearly perfect conservation of the amino acid loop in higher eukaryotes. Remarkably, we identified two amino acids in the loop of CNMM3 that were perfectly conserved in the entire analysis: aspartic acid 426 and proline 427 (Fig. 1*A*). Thus, we wanted to further characterize the binding affinities of all residues present on the extended loop by mutating each of the amino acids from 424 to 429 by alanine to identify the critical residue(s) involved in the complex formation. We examined their binding abilities by co-transfecting GST-PRL-2 and FLAG-2xCBS (Bateman module) mutants of CNNM3 in HeLa cells, followed by FLAG immunoprecipitation. Interaction was completely lost when the 2xCBS mutants D426A, P427A and Y429A were independently expressed (Fig. 1*B*). Interestingly, the 2xCBS T436I mutation corresponding to the T568I mutation in CNNM2, shown to cause hypomagnesemia in human (18), also reduced PRL-2 binding. The importance of the two most conserved amino acids (Asp-426 and Pro-427, identified in Fig. 1*A*) was further confirmed by co-transfecting GST-PRL-2 and the full-length CNNM3 mutants in HeLa cells, followed by GST pulldown (Fig. 1*C*). From those findings, we selected the Asp-426 mutant to continue our investigation, given that charged residues are known to promote high-affinity protein binding (27, 28) and because of the very high conservation of Asp-426 in other eukaryotic CNNMs. Because we demonstrated previously that disturbing the full structure of the Bateman module using the CNNM3 G433D mutant was able to alter the interaction with PRL-2 (5), we compared its effect with that of the D426A mutant and showed that they both equally decrease the binding with PRL-2 (Fig. 1*D*). Importantly, this aspartic acid was further mutated to vary both the length and the charge of the residue to determine the necessity of aspartic acid at this position in the loop to mediate the interaction with PRL-2 (Fig. 1*E*).

We recently found that mutations of essential catalytic residues in PRL-2, like D69A or C101S, also cause loss of interaction with CNNM3, suggesting that residues within the catalytic cavity are critical for complex formation (5). Prompted by these findings and our mutagenesis of the Bateman module described above, we scrutinized the interactions underlying the human PRL-2·CNNM3 assembly by molecular modeling approaches. Using the ZDOCK protein-protein docking prediction program (24), we established the top 10 possible docking orientations of the complexes that could be grouped in three different types of arrangement (supplemental Fig. 2). The highest scored was the one that proposes a direct interaction between the extended loop of CNNM3 and the catalytic cavity of PRL-2 (Fig. 1*F*). According to this model, the two proteins associate through electrostatic interactions participated in by three acidic residues, Glu-422, Glu-424, and Asp-426, of the extended loop (Fig. 1, *B* and *F*). Interestingly, the side chain of Asp-426 buries into the catalytic cavity of PRL-2 and occupies a nearby position of the phosphatase potential substrates. Two alternating glycines, located at positions 423 and 425, allow the acidic residues to face the surface of PRL-2. On the contrary, the N- and C-terminal residues of the loop (Asn-420 and Phe-428) point toward the outside of the catalytic cavity, suggesting a secondary role in the interaction. Taken together, our findings establish the importance and specificity of aspartic acid 426 of CNNM3 for binding with PRL-2 to form a stable protein complex.

##### Functional Characterization of the CNNM3 WT and D426A Mutant

**FIGURE 2.**
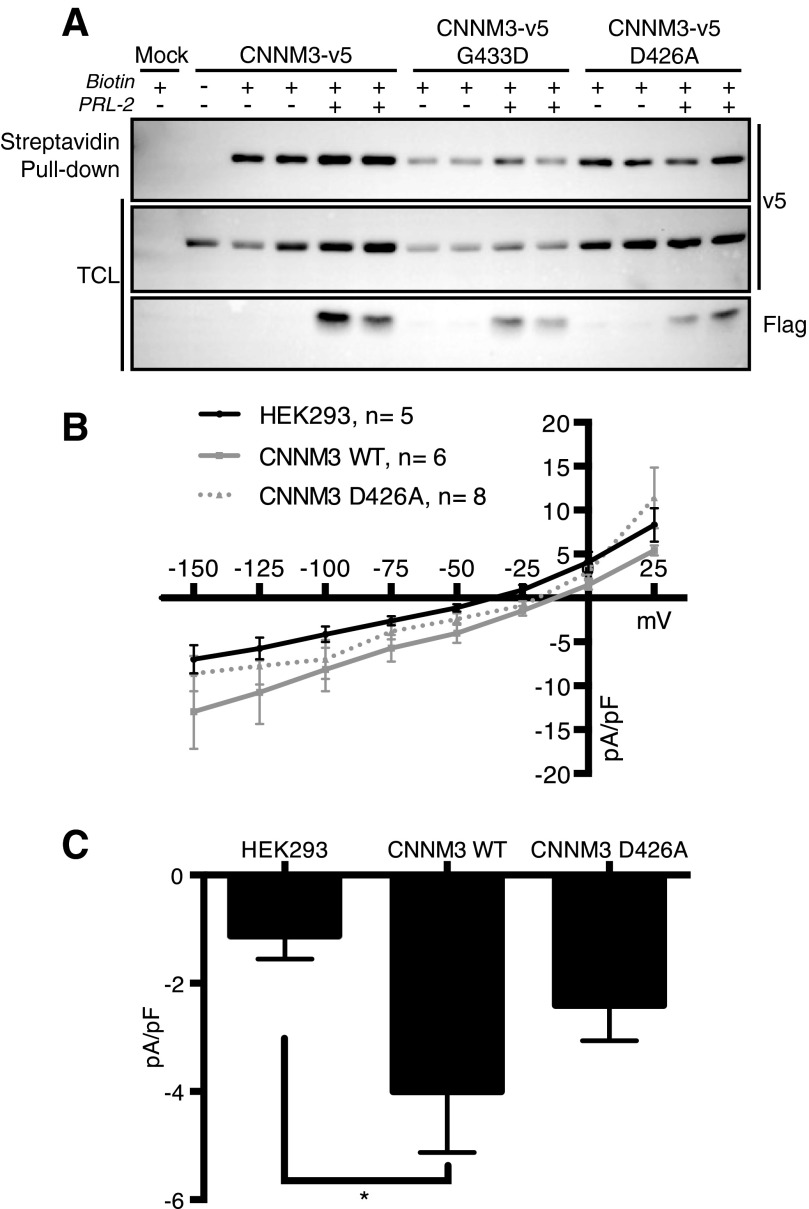
**Functional characterization of CNNM3 WT and the D426A mutant.** *A*, HEK293 cells were transiently co-transfected with CNNM3-v5 and FLAG-PRL-2 constructs. The cell surface was biotinylated, and the cell extract was subjected to a streptavidin-agarose pulldown and immunoblot analysis. *TCL*, total cell lysate. *B*, current-voltage relationship was obtained from voltage clamp protocols that involved a holding potential of 0 mV from which 2 s steps were made in increments of 25 mV over the range of −150 mV to +25 mV, followed by a return to 0 mV (*n* = 5, *black*; CNNM3-WT-transfected, *n* = 6, *gray*, and CNNM3-D426A-transfected HEK293 cells, *n* = 8, *dashed*). *pF*, picofarad. *C*, quantitative measurement of current magnitude from the three groups represented in *B* at the physiological epithelial voltage of −50 mV. All data are expressed as mean ± S.E. *, *p* = 0.0322 by one-way ANOVA.

CNNM2 has been shown to reside at the plasma membrane, and membrane topology studies showed that CNNM2 has an extracellular N terminus plus an intracellular C terminus that contains the Bateman module (16). Accordingly, to determine the membrane expression of the CNNM3 mutant that has lost its ability to interact with PRL-2, cell surface biotinylation assays of the CNNM3 WT-v5 and CNNM3 D426A-v5 mutants overexpressed in HEK293 cells in the presence of FLAG-PRL-2 were performed (Fig. 2*A*). The presence at the plasma membrane of the CNNM3 WT and mutant correlates with their levels of ectopic expression and was not affected by the presence of PRL-2. This suggested that neither the mutations nor PRL-2 affect CNNM3 membrane stability and levels at the cell surface.

To examine the influence of CNNM3 on magnesium transport, we investigated the functional characteristics of CNNM3 by transfecting CNNM3 WT or CNNM3 D426A into HEK293 cells and electrophysiologically recorded these cells using whole-cell patch clamping. Fig. 2*B* shows that CNNM3 WT tends to shift the whole-cell macroscopic current-voltage relationship in the inward direction relative to that of non-transfected cells. The current-voltage relationship of CNNM3 D426A fell between those of CNNM3 WT and non-transfected cells. Importantly, when current magnitude was measured at the epithelial physiological resting potential of −50 mV (Fig. 2*C*), there was a significantly larger inward current in cells transfected with CNNM3 WT compared with non-transfected cells. In contrast, the currents recorded from cells transfected with CNNM3 D426A were not significantly different from non-transfected cells, suggesting that the binding of PRL-2 to CNNM3 is important for the function of the complex.

##### Disruption of the PRL-2/CNNM3 Complex Decreases Cell Proliferation and Tumors

**FIGURE 3.**
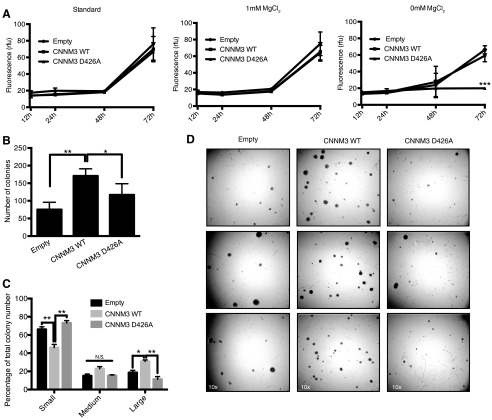
**Overexpression of CNNM3 D426A in DB-7 mammary cancer cells decreases proliferation.** *A*, proliferation assays of DB-7 stable lines expressing pLenti6/v5 (*Empty*), pLenti6/v5 CNNM3 WT (*CNNM3 WT*), and pLenti6/v5 CNNM3 D426A (*CNNM3 D426A*) was performed using CyQUANT DNA dye after culturing the cells with standard growth medium (*left panel*), magnesium-free medium supplemented with 1 mm MgCl_2_ (*center panel*), or without supplementation (*right panel*). Values are mean ± S.D. ***, *p* = <0.0001 by two-way ANOVA followed by multiple comparison test. *B* and *C*, DB-7 stables lines were tested for their potential to form colonies in soft agar by quantifying the number of colonies (B) and the percentage of small (50 to <500 pixels), medium (500 to <1000 pixels), and large (≥1000 pixels) colonies (*C*), normalized to total colony number per group. Values are mean ± S.E. *, *p* = 0.0039; **, *p* = <0.0001; *N.S.*, not significant by one-way ANOVA followed by multiple comparison test. *D*, representative pictures of soft agar assays from DB-7 stable lines at ×10 magnification.

The regulation of intracellular magnesium levels by PRLs in cancer has been proposed previously to be mediated by its interaction with the CNNMs magnesium transporter family (5, 9), but the structural basis of this interaction is still obscure. To assess the oncogenic potential of the PRL-2·CNNM3 complex, we first investigated the role of the CNNM3 D426A mutant, which has lost its ability to interact with PRL-2, in cellular proliferation by establishing cell lines stably expressing an empty control vector (pLenti6/v5), CNNM3 WT, or CNNM3 D426A in mouse DB-7 tumor-derived cell lines known to form tumors when injected into murine mammary fat pads (3, 29). Although there was no difference in cellular proliferation between empty vector-, CNNM3 WT-, and D426A-expressing cells using standard medium (Fig. 3*A*, *left panel*), we hypothesized that the loss of interaction with PRL-2 might have a disadvantageous effect in a more stringent environment. Because the CNNM3/PRL-2 interaction is promoted by the presence of low extracellular magnesium levels (5), we tested the ability of the stable cell lines to proliferate in magnesium-deprived medium (Fig. 3*A*, *center* and *right panels*). Interestingly, we found that only the overexpression of CNNM3 D426A impaired the ability of the cell to grow in magnesium-depleted medium (Fig. 3*A*, *right panel*), suggesting a critical role for the PRL-2·CNNM3 complex in magnesium-dependent proliferation.

To further evaluate the effect of this interaction on the tumorigenic capacity of the cells, we subjected the DB-7 stable cell line to anchorage-independent growth, which is a hallmark of a transformed phenotype (30, 31). We observed that overexpression of CNNM3 WT favored colony formation (Fig. 3*B*) and that it had an increased proportion of large colonies (Fig. 3*C*) compared with both control and D426A mutation. Thus, the data support an essential role for PRL-2 in CNNM3-dependent cell proliferation in a stringent environment.

**FIGURE 4.**
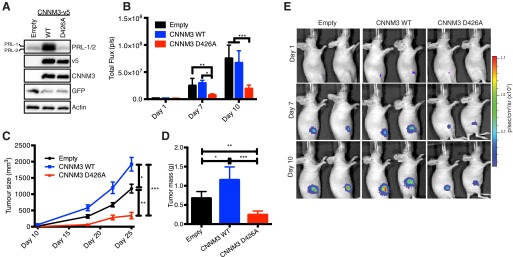
**Disruption of the PRL-2·CNNM3 complex formation decreases tumor growth in a xenograft mouse model.** *A*, mice (NU/J, 6 weeks old) were injected in the mammary fat pad with DB-7 mammary cancer cells stably expressing CNNM3 WT or the CNNM3 D426A mutant and a luciferase reporter gene. *B*, after injection, tumor sizes were monitored by luciferase bioluminescence through total flux (photons/second) *, *p* = 0.0447; **, *p* = 0.0043; ***, *p* = <0.0001. *C*, caliper measurements were taken when the tumors were visible. Values are mean ± S.D. *, *p* = 0.0122; **, *p* = 0.0057; ***, *p* = <0.0001 by two-way ANOVA followed by multiple comparison test. *D*, tumor were dissected out at the end point and weighed. Values are mean ± S.D. *, *p* = 0.0104; **, *p* = 0.0046; ***, *p* = <0.0001 by one-way ANOVA followed by multiple comparison test. *E*, representative picture from all groups in the first 10 days (*n* = 8/group).

To translate the importance of our findings to an *in vivo* context, we used an orthotopic xenograft model that closely mimics tumor microenvironments, including magnesium availability. Thus, to assess the oncogenic capability of the CNNM3 D426A mutant that lost its ability to interact with PRL-2, we generated stable mouse mammary tumor DB-7 cells (3) co-overexpressing CNNM3 WT-v5 or CNNM3 D426A-v5 (Fig. 4*A*) and luciferase, which were injected in the mammary fat pads of athymic nude female mice. The luciferase activity of those cell lines was measured prior to injection and was similar for all conditions (data not shown). A bioluminescence imaging system also confirmed a similar luciferase signal of the DB-7 stable cell lines present at the injection site. Early after tumor injection, we observed lower luciferase activity in cells overexpressing CNNM3 D426A, which was consistent on days 7 and 10 (Fig. 4, *B* and *E*). Remarkably, cells overexpressing the CNNM3 D426A mutant presented reduced tumor size compared with an exponential growth for both empty control and wild-type protein (Fig. 4*C*). Moreover, the tumors were dissected at the experimental end point, and we similarly observed that cells overexpressing the mutant had a lower tumor burden (Fig. 4*D*). Together, these results demonstrate that the ability of CNNM3 to interact with PRL-2 is essential for tumor progression and that blocking PRL-2·CNNM3 complex formation *in vivo* causes a growth disadvantage for the tumor cells.

##### The PRLs Inhibitor Thienopyridone Blocks the Interaction between PRL-2 and CNNM3 to Reduce Cancer Cell Proliferation

**FIGURE 5.**
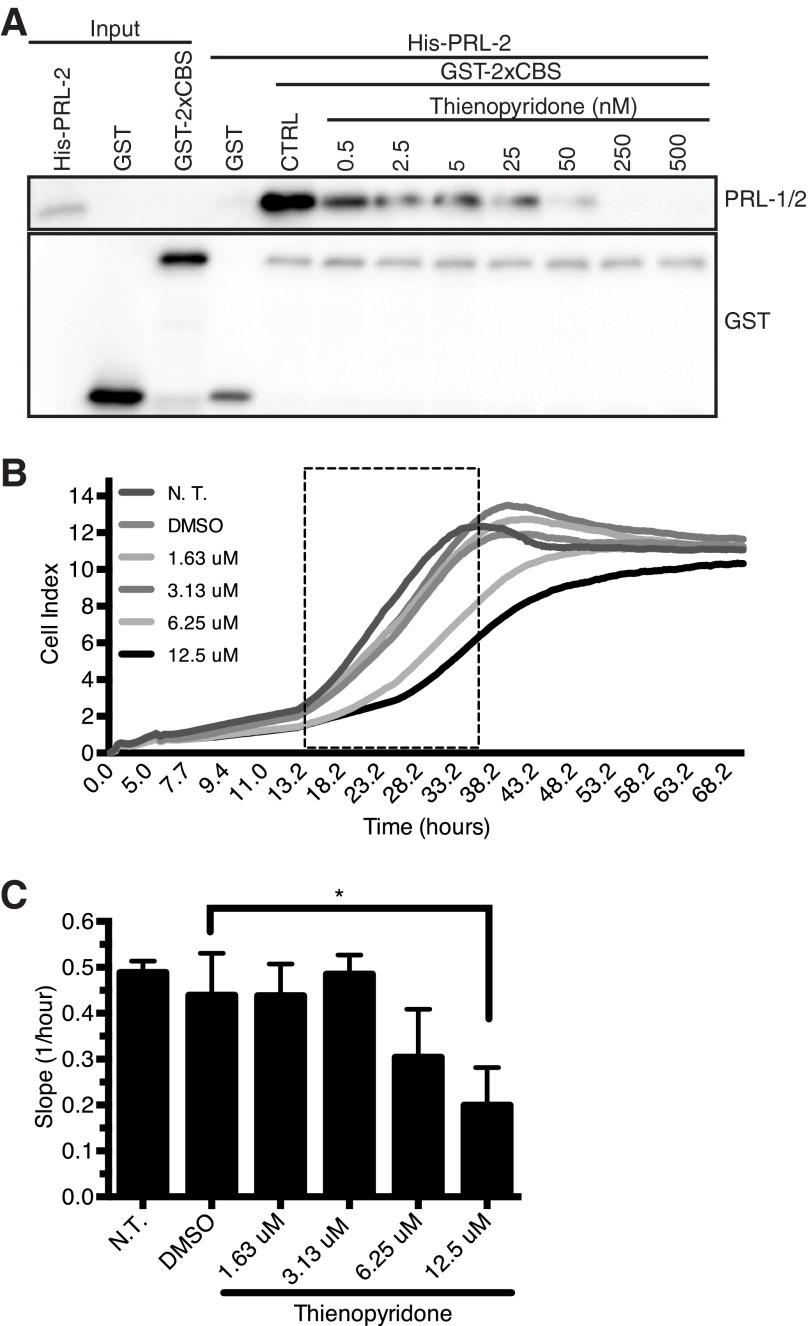
**Thienopyridone blocks the interaction between PRL-2 and the Bateman module of CNNM3 to reduce proliferation.** *A*, *in vitro* interaction of PRL-2 with the CBS tandem domain of CNNM3. His-PRL-2, GST-2xCBS and GST alone were produced in bacteria. GST pulldown was performed with equimolar amounts of both proteins with various concentrations of thienopyridone. The control (*CTRL*) represents the binding between 2xCBS and PRL-2 without inhibitor. *B*, real-time analysis of the effect of thienopyridone on MCF-7 cell using the xCELLigence system. The *dotted box* represents the linear phase of cell proliferation. *DMSO*, dimethyl sulfoxide; *NT*, not treated. *C*, the slope (1/h), representing the cell ability to proliferate was calculated based on the cell index values in the linear phase of the plot (*dotted box*) described in *B*. Each drug condition was done in triplicate, and the results are represented as mean ± S.D. *, *p* = 0.0231 by one-way ANOVA followed by multiple comparison test. Dimethyl sulfoxide was used as a vehicle control.

Our CNNM3·PRL-2 modeling indicates that the side chain of the Asp-426 residue buries into the catalytic cavity of PRL-2 and occupies a position near the phosphatase potential substrate site (Fig. 1*F*). Thus, we speculated that a competitive inhibitor of PRL-2 could abrogate the interaction with CNNM3 and, according to our results described above, the oncogenic activity of the complex. Thienopyridone is a known specific inhibitor of PRLs that was shown previously to impair their enzymatic activities and decrease both the proliferation and migration abilities of cells overexpressing PRLs (32, 33). We first investigated the effect of this small inhibitor on the binding of recombinant PRL-2 and the Bateman module of CNNM3 *in vitro* by performing a pulldown experiment in the presence of various concentrations of inhibitor. In this assay, thienopyridone was able to disrupt the binding between PRL-2 and CNNM3 (Fig. 5*A*). Because MCF-7 human breast cancer cells expressed both PRL-2 and CNNM3 (5), we tested their proliferation rate with various concentrations of thienopyridone using the xCELLigence system (Fig. 5*B*) and showed that this small molecule inhibitor was able to decrease cell proliferation (Fig. 5*C*). These results strongly suggest that the mechanism by which this inhibitor impairs cellular growth is through inhibition of the PRL-2 interaction with CNNM3.

## Discussion

In this study, we identified a highly conserved aspartic acid located on the extended loop of the Bateman module of CNNM3 critical for the binding with PRL-2. This observations is consistent with the fact that most protein-protein interactions are mediated by non-helical, non-strand peptide loops (34). Using a CNNM3 mutant (D426A) that has lost its capacity to interact with PRL-2 or a small molecule inhibitor of the complex formation between these two proteins, we showed that this interaction is important for breast cancer cell proliferation and tumor growth.

PRL-2 expression was shown previously to correlate with cancer progression (3, 35), and PRLs were demonstrated recently to interact with CNNMs to promote oncogenesis by regulating intracellular levels of magnesium (5, 9). Here we characterized more precisely the interaction using various point mutants that lead to the modeling of the interaction using previously published structures of the Bateman module of CNNM2 and PRL-1 (36, 37). The modeled structure of the interaction in Fig. 1*F* shows that amino acid 426 of CNNM3 binds in the catalytic site of PRL-2, which interferes with potential substrate binding. These results confirm our previously proposed model that CNNM3 is not a substrate of PRL-2 (5). The PRL phosphatases are known to be poorly active, using *in vitro* enzymatic assays with synthetic substrates, and the few putative substrates remain to be confirmed (5–7). Thus, this can explain the difficulty for identifying PRL-2 physiological substrates if the major role of this PTP resides upon binding with CNNM3 to regulate magnesium transport. On the other hand, this does not exclude potential CNNM3 or PRL-2 mutually exclusive roles.

The structural modeling also suggests that using an inhibitor targeting the catalytic pocket of PRLs would inhibit its interaction with CNNMs. Thienopyridone has been shown to selectively inhibit PRL activity compare with other PTPs and also had an antiproliferative effect in cancer cells (32). Although this effect on cell growth was associated with the loss of PRL activity, we speculated that it could potentially be caused by the disruption of the PRL·CNNM complex. Here we showed that indeed this molecule blocked the *in vitro* interaction of PRL-2 with CNNM3 and to also reduced MCF-7 breast cancer cell proliferation, thus corroborating our hypothesis. This experiment creates an opportunity to further investigate the effect of PRL inhibitors that have been developed recently (33) on PRL-CNNM complex formation. Moreover, it is a proof of concept to further explore the possibility of blocking this interaction for clinical purposes in cancer therapy.

We previously uncovered that the loop region of the second CBS of the Bateman module of CNNMs and PRL phosphatases appear at the same stage during evolution (5). Interestingly, here we showed that specific amino acids of this region are almost fully conserved, and when we mutated them in CNNM3, they lost the interaction with PRL-2. Moreover, a point mutation of the conserved aspartic acid in this loop is sufficient to block complex formation, resulting in reduced tumor growth. The CNNM3 D426A mutant has a dominant negative effect on cancer cell proliferation in magnesium-deprived medium. Consistent with this, a previous observation showed that low magnesium promotes the PRL-2/CNNM3 association (5). Thus, blocking this association will severely hinder the ability to proliferate when magnesium is less available. Because cancer cells have to adapt to survive the harsh tumor microenvironment (38), this could explain the mechanistic basis for the high expression levels of PRLs observed in various cancers.

Although electrophysiological studies have been previously performed on CNNM2, the mechanism of action of this subfamily of magnesium transporter remains poorly characterized because published results remain ambiguous. Stuiver *et al.* (18) showed than an increase in extracellular Mg^2+^ blocked CNNM2 influx. In contrast, Quamme (14) observed the opposite, where high extracellular Mg^2+^ promotes CNNM2 current. To date, CNNM2 is the only member of the CNNM family to be tested by patch clamping. To clarify the mechanism of action of CNNM3, we examined its propensity to alter voltage gating. Our novel electrophysiological analysis indicates that the expression of CNNM3 increased the cellular current under hyperpolarization conditions, as similarly observed for CNNM2 (18). Moreover, because the T568I mutation in the second CBS domain of CNNM2 has been described in patients with dominant hypomagnesemia to result in a smaller Mg^2+^-sensitive current when overexpressed in HEK293 cells (18) and because the T568I mutation in CNNM2 is known to disrupt the binding with Mg^2+^/ATP (9, 16, 39), we also tested the corresponding (T436I) threonine in CNNM3. Here we observed a decrease in binding of PRL-2 when this corresponding threonine was mutated in CNNM3, suggesting a possible role for PRL in this CNNM-associated genetic disease. Concomitantly, we also did not detect any changes in cell current in the presence of CNNM3 D426A, strongly indicating that the contribution of PRL-2 binding in the complex is essential for CNNM3-induced cellular current that will lead to increase tumor growth. In support of this view, it is well established that cancer cells proliferate in a state of hyperpolarized membrane (40), which correlates with our findings with CNNM3 at −50 mV. Overall, those findings support the view that the mechanism of action of CNNMs requires the binding of PRLs to trigger a rise in intracellular magnesium levels, as proposed previously (5, 9).

Here the overexpression of both the CNNM3 wild type or binding mutants was found at the plasma membrane independent of PRL-2 expression, suggesting that, although its binding affects magnesium transport, its presence does not influence CNNM3 localization. Also, CBS-PRL complex modeling indicates (Fig. 1*F*) that a single point mutation of the aspartate does not severely disturb the folding of CNNM3 because the mutant was properly localized at the membrane. Similarly, the T568I point mutation in the second CBS of CNNM2 was also correctly directed to the plasma membrane (18). On the other hand, in chloride channels from the CLC family, the presence of structurally functional CBS domains has been shown to influence, either directly or indirectly, their subcellular localization (41). Still, using confocal microscopy, Hirata *et al.* (42) showed that deletion of the Bateman domain in CNNM2 and CNNM4 similarly did not affect their presence at the plasma membrane.

The PRL phosphatases share high sequence identity with each other, and the CBS domains of CNNMs are also highly conserved (5, 43). Nevertheless, different PRL-CNNM complexes were reported to have different ways of regulating magnesium transport but similar consequential effects on intracellular magnesium levels. Our group showed previously that the PRL-2·CNNM3 complex promotes breast cancer progression by regulating magnesium influx (5). On the other hand, Funato *et al.* (9) suggested that PRL-3 binding to CNNM4 blocked its efflux activity to promote colon cancer development. Despite these differences, the consequences of both proposed models lead to an increased intracellular magnesium concentration. In addition, CNNM2 activity was suggested previously to regulate the TRPM7 magnesium channel (19) that is associated with breast cancer growth and metastasis (44, 45), which may also provide a third model of magnesium regulation in cancer by the PRL-CNNM complex. Further molecular, structural, and biological studies are needed to decipher the exact roles of each PRL-CNNM complex. Still, not only is this mechanism novel, but the PRL-dependent magnesium level modulation could also explain the observations relating to the array of signaling pathway activation reported in the past decade in response to PRL overexpression (1, 3, 6). For instance, it was observed recently that overexpression of PRL-3 promotes mechanistic target of rapamycin phosphorylation (46) and that this enzyme is critical for the control of mRNA translation (47). Because both protein phosphorylation and mRNA translation are strongly dependent on magnesium availability (48–50), it is reasonable to speculate that the action of PRLs is to provide enough magnesium for those processes via complex formation with the CNNMs, and this could explain the increase in mechanistic target of rapamycin activity observed in this study.

Together, our data unveiled the importance of the highly conserved amino acid loop present in CNNMs toward modulating their binding to PRLs. Furthermore, we showed that this binding is crucial to the oncogenic activity of PRLs in various types of tumors. We trust that, by inhibiting the formation of PRL-CNNM complexes, we are opening new avenues in cancer therapy suitable to a large array of human cancer types where PRLs are overexpressed.

## Author Contributions

E. K. conducted most of the experiments and analyzed the results. W. C. V. and A. S. designed and conducted the experiments on CNNM3 surface current function. A. K., J. H. F. d. B., and J. G. J. H. designed and conducted the experiments on CNNM3 biotinylation. Y. Z. conducted the experiment on Bateman module conservation. M. L. conducted the experiment on MCF-7 proliferation with the PRL inhibitor. N. U. made the initial observation of a PRL link with magnesium levels. L. A. M. C. designed and conducted the CNNM3·PRL-2 complex modeling. E. K. and S. H. wrote the manuscript, and S. H. provided critical advice on the experimental protocol and revisions of the manuscript. M. L. T. conceived the idea of the project, provided all resources and funding, and edited the manuscript.

## Supplementary Material

Supplemental Data

## Footnotes

2 The abbreviations used are:

PTP: protein tyrosine phosphatase
PRL: phosphatase of regenerative liver
CNNM: cyclin M
CBS: cystathionine β synthase
ANOVA: analysis of variance.
