# Supplementary material for: Inhibition of PRL-2·CNNM3 Protein Complex Formation Decreases Breast Cancer Proliferation and Tumor Growth
Source: J Biol Chem. 2016 Mar 11;291(20):10716–25. doi: 10.1074/jbc.M115.705863 (PMC4865918; doi:10.1074/jbc.M115.705863)
Supplement: Supplemental Data [file supp_291_20_10716__index.html]

Inhibition of the PRL-2/CNNM3 Protein Complex Formation Decreases Breast Cancer Proliferation and Tumor Growth. — Inhibition of PRL-2·CNNM3 Protein Complex Formation Decreases Breast Cancer Proliferation and Tumor Growth — CNNM3 Is Required for PRL-2 Oncogenic Activities — Supplemental Data 

# Inhibition of PRL-2·CNNM3 Protein Complex Formation Decreases Breast Cancer Proliferation and Tumor Growth

## Supplemental Data

- Supplemental Figures (.pdf, 802 KB) - Suppl. Fig 1: Conservation of Bateman module in 150 eukaryotic sequences. Suppl. Fig 2: Protein-protein prediction models.
