# Supplementary material for: Inhibition of PRL-2·CNNM3 Protein Complex Formation Decreases Breast Cancer Proliferation and Tumor Growth
Source: J Biol Chem. 2016 Mar 11;291(20):10716–25. doi: 10.1074/jbc.M115.705863 (PMC4865918; doi:10.1074/jbc.M115.705863)
Supplement: Supplemental Data [file 10.1074_M115.705863_jbc.M115.705863-1.pdf]

Inhibition of the PRL-2/CNNM3 protein complex formation decreases breast cancer proliferation and tumor growth.

Elie Kostantin[1,2], Serge Hardy[1], William C. Valinsky[3], Andreas Kompatscher[4], Jeroen H. F. de Baaij[4], Yevgen Zolotarov[1,2], Melissa Landry[1], Noriko Uetani[1], Luis Alfonso Martínez-Cruz[5], Joost G. J. Hoenderop[4], Alvin Shrier[3], and Michel L. Tremblay[1,2]

From the [1]Rosalind and Morris Goodman Cancer Research Centre, Montréal, Canada, the [2]Department of Biochemistry, McGill University, Montréal, Canada, the [3]Department of Physiology, McGill University, Montreal, Canada, the [4]Department of Physiology, Radboud Institute for Molecular Life Sciences, Radboud University Medical Center, Nijmegen, The Netherlands, and the [5]Structural Biology Unit, Center for Cooperative Research in Biosciences (CIC bioGUNE), Technology Park of Bizkaia, Spain

Running Title: CNNM3 is required for PRL-2 oncogenic activities.

To whom correspondence should be addressed: Prof. Michel L. Tremblay, Rosalind and Morris Goodman Cancer Research Centre, McGill University, 1160 Pine Avenue W, Montreal, Quebec, Canada H3A 1A3, Phone: (514) 398-7290, Fax: (514) 398-6769, E-mail: michel.tremblay@mcgill.ca

Keywords*:* phosphatase, cancer, cell proliferation, magnesium, patch clamp, PRL-2, CNNM3, Thienopyridone, Bateman module

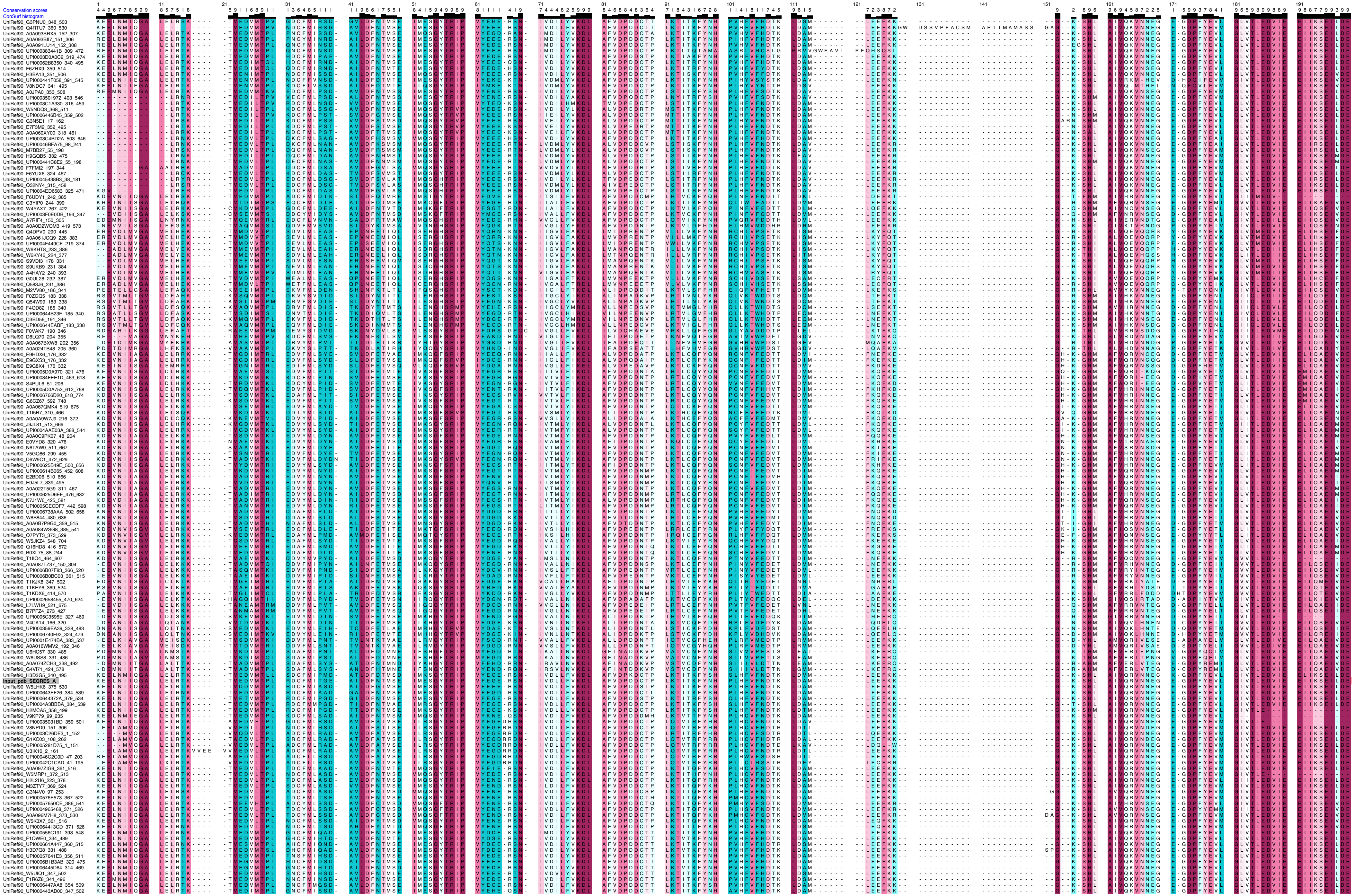

SUPPL. FIGURE 1: Conservation of Bateman module in 150 eukaryotic sequences. The Bateman module of CNNM2 was used to determine the conservation of its Bateman module among 150 eukaryotic sequences using ConSurf. The CNNM2 sequence is identified in the list as input and is higlighted. The sequences are colored based on the residue conservation ranging from cyan (lowest) to magenta (highest).

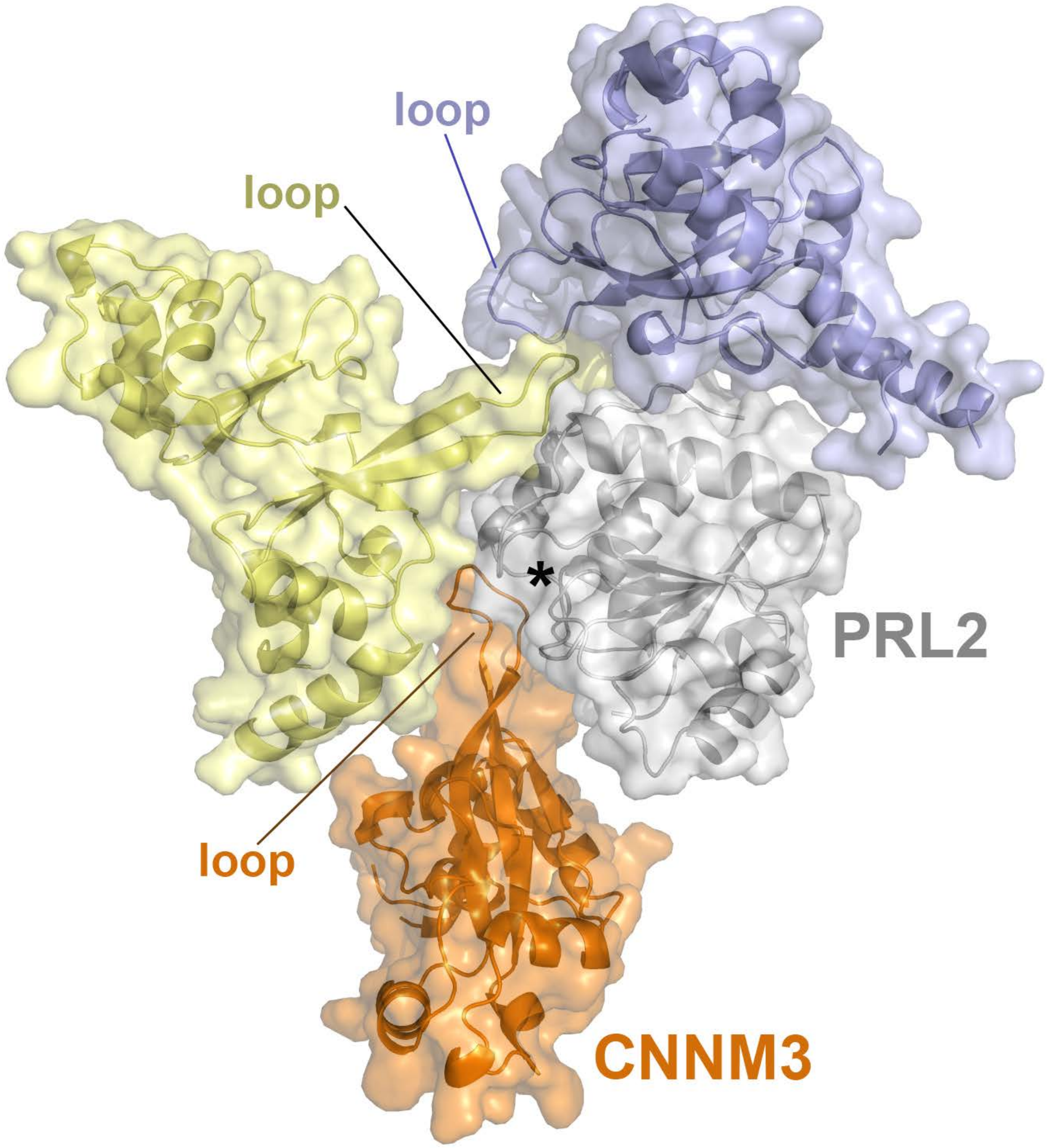

SUPPL. FIGURE 2: Protein-protein prediction models. The figure represents three different CNNM3Bat+PRL2 arrangements predicted by ZDOCK. The Bateman module of CNNM3 is represented in orange, yellow and blue. The highest score model, which places the extended loop of the Bateman module of CNNM2 (orange) interacting with residues of the catalytic cavity of PRL2 (in grey). The lower score models (in blue and yellow) are not consistent with former experimental data. The asterisk indicates the position of substrates in PRL2.
